# Quality of Life of Autistic Children and Supported Programs in Saudi Arabia: A Cross-Sectional Study

**DOI:** 10.7759/cureus.51645

**Published:** 2024-01-04

**Authors:** Rahaf Mohammed Alasiri, Danah Abdullah Albarrak, Deemah Meshal Alghaith, Ohoud Saad Alsayari, Yasmeen Salem Alqahtani, Ammar Y Bafarat, Noha Farouk Tashkandi

**Affiliations:** 1 Medicine, King Saud Bin Abdulaziz University for Health Sciences College of Medicine, Riyadh, SAU; 2 Psychiatry, King Abdulaziz Hospital, Jeddah, SAU; 3 Psychiatry, Ministry of National Guard Health Affairs, Alahsa, SAU; 4 Medical Research, King Saud Bin Abdulaziz University for Health Sciences College of Medicine, Riyadh, SAU

**Keywords:** patients’ satisfaction, cross-sectional study, kingdom of saudi arabia (ksa), autism spectrum disorder (asd), quality of life

## Abstract

Background: Autism spectrum disorder (ASD) is a neurobehavioral condition marked by social interaction challenges, communication deficits, and repetitive behaviors, with studies in Saudi Arabia showing varying prevalence rates in different regions. This study aimed to evaluate the social context of autistic children and the quality of life (QOL) for families of children with autism utilizing the Beach Center Family Quality of Life Scale (BCFQOL).

Methods: This cross-sectional study, conducted at King Abdulaziz Medical City in Saudi Arabia over a six-month period, included ASD children aged 1-14 years. The QOL was measured using the BCFQOL scale for families. Data were analyzed using the Jamovi software (Windows version 2.4.1, the Jamovi Project, retrieved from https://www.jamovi.org).

Results: A total of 102 responses were collected in the study. The overall satisfaction score was 93.6±16.6 out of 125, with 85.3% of participants expressing satisfaction. Domains explored included family interaction (23.8±5.29 out of 30), parenting practices (23.9±3.83 out of 30), emotional well-being (13.1±4.16 out of 20), physical and material well-being (18.7±4.24 out of 25), and disability-related support (14.2±4.0 out of 20). In terms of specific sociodemographic factors, no statistically significant differences in satisfaction were observed across various categories.

Conclusion: Families of children with ASD in Saudi Arabia generally report high levels of satisfaction, as assessed by the BCFQOL. The study covered various domains, including family interaction, parenting practices, emotional well-being, physical well-being, and disability-related support, with most respondents expressing satisfaction in these areas. Notably, sociodemographic factors did not significantly influence satisfaction levels, underscoring the pervasive nature of the findings across different demographic groups. Further studies with a larger sample size and a longer follow-up period are required to validate these findings.

## Introduction

Autism spectrum disorder (ASD) includes a variety of neurobehavioral disorders [1]. These are typically marked by challenges in social interaction, communication deficits, and narrow interests that are often repetitive in nature [2]. Most diagnoses of ASDs occur early in a child's life, usually within the initial three years, and persist into adolescence and adulthood [3]. The World Health Organization indicates that around one in 160 children displays ASD-related symptoms, with a rising global prevalence [2]. In Saudi Arabia, a cross-sectional study conducted in Riyadh with children aged two to four reported a prevalence of 2.51%, 25 per 1000, with a 3:1 female-to-male ratio [4]. In another cross-sectional study of the prevalence of ASDs in Makkah and Jeddah, the rate was 2.618 per 1,000 in Jeddah, 3.68 per 1,000 in Makkah, and 2.81 per 1,000 in both Jeddah and Makkah [5].

No specific cure for ASD exists; however, treatments aiming to enhance the quality of life (QOL) for those with ASDs are available. These treatments rely on early interventions and consistent developmental monitoring by caregivers [2]. Caregivers, often family members or designated individuals, are tasked with supporting dependents, including children or those with health- or age-related challenges [6]. Particularly, caregivers for children with ASDs provide multifaceted care, from physical and psychological to social, which often surpasses the support levels rendered to non-ASD children [7]. The demands of catering to a child with ASD can be intense. Compounded by the challenges of accessing relevant services and support, caregivers' QOL can diminish. Hence, equipping caregivers becomes pivotal in ASD care interventions [2].

Global studies underscore the challenges of caregiving for children with ASD [8,9]. A 2018 US study revealed that mothers of children with autism face multifarious obstacles during recreational activities, such as limited personal time, intensified supervisory requirements, and issues securing suitable assistance [9]. In the Arabian Gulf countries, child healthcare provisions in this context remain inadequate [10]. However, a 2012 study from Qatar emphasized the substantial impact of caregiving on QOL and proffered recommendations like bolstered support for children with ASDs and their caregivers [8]. One study conducted in the eastern region of Saudi Arabia found that the most pressing challenge associated with raising an autistic child was the lack of awareness among the general public [11]. The second challenge faced by the families was finding autism centers [12]. This highlights the need for raising awareness and providing appropriate centers specializing in children with autism. Furthermore, autism is associated with minimal verbal communication at least until the age of five [13,14]. Some of these children can benefit from interventions that increase their verbal communication abilities [15]. In order to provide effective communication interventions for children with autism who are minimally verbal, peers with no disabilities must be trained to be more effective communicators with these children [16].

Studies indicate that early diagnosis and intervention of ASD may result in better outcomes, particularly for children under two years of age [17]. Unfortunately, many children don't receive a diagnosis until they are much older. A recent study conducted in Saudi Arabia revealed a mean age of 3.8 years at the time of the first diagnosis of ASD [18]. Besides, children from families residing in other countries were more likely to be diagnosed earlier due to easier access to diagnostic services [9]. According to a study conducted in Jeddah, there are severe deficiencies in autism referrals and services [19]. There is, however, an urgent need for autism services to be made available in order to provide appropriate rehabilitation to children with autism and improve their QOL. In fact, families served by the Saudi Autism Society have reported a significant improvement in their QOL [20].

Several intervention programs for ASD are being provided in Saudi Arabia, including the Treatment and Education of Autistic and Related Communication Handicapped Children (TEACCH) program, as well as applied behavior analysis (ABA). Interestingly, speech therapy was found to be the most common intervention utilized by children with autism [18]. It should be noted, however, that some programs are only available in major cities or healthcare organizations [18]. For instance, ABA is recognized internationally as a standard intervention, but in Saudi Arabia, it is only provided by two organizations with ABA-certified therapists [21]. In addition, the TEACCH program is the only intervention that is being used for autistic children of school age. However, this program is not appropriate for all autistic patients, especially those with a high level of functionality [21]. Given the scarce evidence on the effectiveness and application of these programs in the Saudi Arabian context, a thorough investigation into their roles and suitability is crucial to provide healthcare stakeholders with the insights needed to effectively support the implementation and adaptation of these programs. This study aimed to evaluate the social context of autistic children and the QOL for families of children with autism utilizing the Beach Center Family Quality of Life Scale (BCFQOL).

## Materials and methods

Study design and settings

This cross-sectional study was conducted over a period of six months, from May to November 2023, at King Abdulaziz Medical City (KAMC), Jeddah, and Riyadh, Saudi Arabia, where children diagnosed with autism frequently visit. The questionnaire was performed as a face-to-face interview. Details regarding ASD diagnosis and severity were extracted from pre-existing medical records. Both the QOL of these children and that of their families were assessed. Questionnaires were distributed to parents or caregivers. One survey focused on assessing the QOL within the family, while the other addressed the child's individual QOL. The study protocol was approved by the Ethics Committee of KAMC (approval number: 1736/23). Written informed consent was obtained from all participants, parents, or family members.

Inclusion and exclusion criteria

We included ASD children aged between one and 14 years, with no restrictions in terms of gender. The diagnosis of ASD was confirmed using the Diagnostic and Statistical Manual of Mental Disorders (DSM-5) [22]. Children who presented with other developmental disorders were excluded. Parents or caregivers must be directly involved in the care of a child diagnosed with ASD to be eligible.

Sampling technique and sample size

A consecutive sampling method was employed to recruit study subjects. With α set at 0.05, β at 0.20, and an anticipated mean of overall satisfaction score of 77.1 [10], it was estimated that the sample must comprise approximately 157 participants for reliable results.

Data collection and instruments

The data metrics include demographics, autism severity indications, and QOL assessments for both children and families. Autism severity was retrieved from the child's medical documentation. The family's QOL was gauged using the accredited Arabic iteration of the BCFQOL scale. This five-point Likert scale involved domains such as family interaction, parenting, emotional well-being, physical/material well-being, and disability-related support.

Statistical analysis

Data were analyzed using the Jamovi software (Windows version 2.4.1, the Jamovi Project, retrieved from https://www.jamovi.org). Categorical variables were presented as frequencies and percentages, while continuous variables, such as satisfaction scores, were presented as the mean and standard deviation (SD). The Chi-square and Fisher's exact tests were performed to explore the association between sociodemographic characteristics and satisfaction. Twenty-five questions assessed satisfaction on a five-point Likert scale, yielding a total score of 125. Satisfaction was classified as poor (≤75 score) or good (>75 score). A p-value less than 0.05 was considered statistically significant.

## Results

Sociodemographic factors

A total of 150 patients were recruited; of them, only 102 responses were collected, with a response rate of 68%. In the study sample, the distribution of participants across the different centers was as follows: KAMC Riyadh (28.4%), KAMC Jeddah (13.7%), online (48.0%), and others (9.8%). The age of the ASD children ranged from 1-4 years (11.8%), 5-7 years (19.6%), 8-10 years (25.5%), 11-13 years (22.5%), and 14-16 years (20.6%). The majority of ASD children were male (76.5%) compared to females (23.5%). In terms of ASD severity scores, 39.2% were classified as Level 1 (mild/requiring support), 48% as Level 2 (moderate/requiring substantial support), and 12.7% as Level 3 (severe/requiring very substantial support). For the ASD children program, the highest percentage of children underwent speech and language therapy (69.6%), followed by behavioral therapy (58.8%), occupational therapy (43.1%), special education programs (38.2%), ABA therapy (25.5%), sensory integration therapy (19.6%), and TEACCH (17.6%).

Relatives' age distribution was divided into <30 years (10.8%), 30-39 years (37.3%), 40-49 years (36.3%), 50-59 years (13.7%), and ≥60 years (2.0%). When looking into the educational background of the relatives, a majority held a college degree (60.8%), followed by high school graduates (28.4%) and those with higher education (10.8%). Occupation-wise, 31.4% were unemployed, 40.2% worked in the government sector, 17.6% in the private sector, 2.0% were students, and 8.8% were retired. The majority of relatives were Saudi (83.3%). The monthly incomes of the relatives were categorized into <10,000 SAR (57.8%), 10,000-20,000 SAR (32.4%), and >20,000 SAR (9.8%). The relation of the relatives to the child was predominantly the mother (59.8%), followed by the father (33.3%), siblings (2.0%), and others (4.9%), as shown in Table 1.

**Table 1:** Sociodemographic and clinical characteristics of included patients SAR: Saudi Arabia Riyal; TEACCH: Treatment and Education of Autistic and Related Communication Handicapped Children; ASD: autism spectrum disorder; KAMC: King Abdulaziz Medical City; ABA: applied behavior analysis

Variables		N (%)
Center	KAMC Riyadh	29 (28.4%)
	KAMC Jeddah	14 (13.7%)
	Online	49 (48.0%)
	Others	10 (9.8%)
ASD children's age group	1-4 years	12 (11.8%)
	5-7 years	20 (19.6%)
	8-10 years	26 (25.5%)
	11-13 years	23 (22.5%)
	14-16 years	21 (20.6%)
ASD children gender	Male	78 (76.5%)
	Female	24 (23.5%)
ASD severity score	Level 1 (mild/requiring support)	40 (39.2%)
	Level 2 (moderate/requiring substantial support)	49 (48%)
	Level 3 (severe/requiring very substantial support)	13 (12.7%)
ASD children program	ABA therapy	26 (25.5%)
	Behavioral therapy	60 (58.8%)
	Speech and language therapy	71 (69.6%)
	Occupational therapy	44 (43.1%)
	Sensory integration therapy	20 (19.6%)
	Special education programs	39 (38.2%)
	TEACCH	18 (17.6%)
Relatives' age	<30	11 (10.8%)
	30-39	38 (37.3%)
	40-49	37 (36.3%)
	50-59	14 (13.7%)
	≥60	2 (2.0%)
Educational level	High school	29 (28.4%)
	Higher education	11 (10.8%)
	College	62 (60.8%)
Occupation	Unemployed	32 (31.4%)
	Government sector	41 (40.2%)
	Private sector	18 (17.6%)
	Student	2 (2.0%)
	Retired	9 (8.8%)
Nationality	Saudi	85 (83.3%)
	Non-Saudi	17 (16.7%)
Monthly income	<10,000 SAR	59 (57.8%)
	10,000-20,000 SAR	33 (32.4%)
	>20,000 SAR	10 (9.8%)
Relation	Mother	61 (59.8%)
	Father	34 (33.3%)
	Siblings	2 (2.0%)
	Other	5 (4.9%)

Satisfaction

The overall satisfaction score was 93.6±16.6 out of 125. Approximately 85.3% of the participants were satisfied/very satisfied (>75 satisfaction score).

Family interaction

In the assessment of family interaction, the total satisfaction score was 23.8±5.29 out of 30. Regarding the enjoyment of family time together, most participants felt "satisfied" (39.2%), closely followed by those feeling "very satisfied" (32.4%). The least satisfied categories were "very dissatisfied" at 4.9% and "dissatisfied" at 10.8%, with a neutral middle ground at 12.7%. When examining open communication within families, the majority felt "satisfied" (39.2%) or "very satisfied" (37.3%). The categories "very dissatisfied" and "dissatisfied" garnered 4.9% and 6.9%, respectively, with 11.8% being neutral. In terms of collaborative problem-solving, the predominant sentiments were "satisfied" (39.2%) and "very satisfied" (31.4%). Minimal families felt "very dissatisfied" (3.9%) or "dissatisfied" (8.8%), and 16.7% were indifferent. When probed about mutual family support toward accomplishing goals, nearly half (47.1%) of participants were "satisfied," and another 32.4% were "very satisfied," whereas dissatisfaction remained minimal (3.9% for "very dissatisfied" and 5.9% for "dissatisfied"). The expression of love and care among family members showed a positive skew, with 45.1% each in the "satisfied" and "very satisfied" brackets, and dissatisfaction was the least at 2.9% for both "very dissatisfied" and "dissatisfied." Lastly, concerning resilience in handling life's vicissitudes, most families felt "satisfied" (48.0%), followed by "very satisfied" (27.5%). A smaller segment felt "dissatisfied" (10.8%) or "very dissatisfied" (2.0%), with a neutral sentiment at 11.8%.

Parenting

In an exploration of parenting practices, the total satisfaction score was 23.9±3.83 out of 30. Regarding fostering independence in children, the bulk of participants felt "satisfied" (53.9%) or "very satisfied" (29.4%). For aiding children with academic endeavors and extracurricular activities, 57.8% were "satisfied," and another 29.4% were "very satisfied." When teaching interpersonal skills, 52.9% felt "satisfied," and 34.3% felt "very satisfied." In guiding children toward making prudent decisions, 52.0% of adults were "satisfied," and 24.5% were "very satisfied." As for adults' familiarity with significant figures in the children's lives, such as friends and teachers, 49.0% expressed being "satisfied" and another 24.5% being "very satisfied." Lastly, in terms of individualized attention and understanding of each child's unique needs, 51.0% were "satisfied," and 20.6% were "very satisfied," although there were some points of contention, with 3.9% being "very dissatisfied" and 11.8% being "dissatisfied."

Emotional well-being

Delving into the domain of emotional well-being, the total satisfaction score was 13.1±4.16 out of 20. For stress relief support, 48.0% of participants were "satisfied," and 16.7% were "very satisfied," though there was a notable segment that was "very dissatisfied" (11.8%). In terms of having friends or external figures to offer support, 36.3% were "satisfied," 17.6% were "very satisfied," and 13.7% expressed strong dissatisfaction. On the topic of finding personal time to pursue individual interests, 38.2% of family members felt "satisfied," and 14.7% felt "very satisfied." However, when considering outside assistance for addressing special needs within the family, sentiments were more polarized. Only 24.5% indicated they were "satisfied," and a mere 10.8% were "very satisfied," juxtaposed against a significant proportion that was "very dissatisfied" (16.7%) or "dissatisfied" (21.6%).

Physical and material well-being

In assessing physical and material well-being, the total satisfaction score was 18.7±4.24 out of 25. When exploring transportation availability, a combined 77.4% felt they had adequate means, with 44.1% being "satisfied" and 33.3% being "very satisfied." Dental care accessibility showed more mixed feelings; while 32.4% were "satisfied" and 20.6% were "very satisfied," a significant 32.3% expressed dissatisfaction. For medical care, a more favorable outlook was observed, with 40.2% of families "satisfied" and 32.4% "very satisfied." When considering financial stability, 42.2% felt "satisfied" with their means to cover expenses, while 21.6% felt "very satisfied." Importantly, feelings of safety across various environments (home, work, school, and neighborhood) were predominantly positive, with 48.0% of respondents "satisfied" and 36.3% "very satisfied."

Disability-related support

When focusing on disability-related support, the total satisfaction score was 14.2±4.0 out of 20. For support at school or the workplace, a combined 55% felt adequately assisted, with 27.5% being "satisfied" and an equal 27.5% being "very satisfied," although 28.4% expressed some form of dissatisfaction. Progress at home had more favorable feedback, with 46.1% of respondents "satisfied" and 27.5% "very satisfied." However, when considering support for their family member with special needs to make friends, the responses were more mixed: while 33.3% were "satisfied," 14.7% were "very satisfied," and 28.4% were not content. Lastly, regarding the relationship with service providers, 41.2% felt "satisfied" with the interactions, and 25.5% felt "very satisfied," but 19.6% had reservations about the quality of the relationship, as shown in Table 2.

**Table 2:** The proportions of satisfaction among patients' families Data are presented with numbers and frequencies

Domain	Item	Satisfaction	N (%)
Family interaction	My family enjoys spending time together	Very dissatisfied	5 (4.9%)
		Dissatisfied	11 (10.8%)
		Satisfied nor dissatisfied	13 (12.7%)
		Satisfied	40 (39.2%)
		Very satisfied	33 (32.4%)
	My family members talk openly with each other	Very dissatisfied	5 (4.9%)
		Dissatisfied	7 (6.9%)
		Satisfied nor dissatisfied	12 (11.8%)
		Satisfied	40 (39.2%)
		Very satisfied	38 (37.3%)
	My family solves problems together	Very dissatisfied	4 (3.9%)
		Dissatisfied	9 (8.8%)
		Satisfied nor dissatisfied	17 (16.7%)
		Satisfied	40 (39.2%)
		Very satisfied	32 (31.4%)
	My family members support each other to accomplish goals	Very dissatisfied	4 (3.9%)
		Dissatisfied	6 (5.9%)
		Satisfied nor dissatisfied	11 (10.8%)
		Satisfied	48 (47.1%)
		Very satisfied	33 (32.4%)
	My family members show that they love and care for each other	Very dissatisfied	3 (2.9%)
		Dissatisfied	3 (2.9%)
		Satisfied nor dissatisfied	4 (3.9%)
		Satisfied	46 (45.1%)
		Very satisfied	46 (45.1%)
	My family is able to handle life's ups and downs	Very dissatisfied	2 (2.0%)
		Dissatisfied	11 (10.8%)
		Satisfied nor dissatisfied	12 (11.8%)
		Satisfied	49 (48.0%)
		Very satisfied	28 (27.5%)
Parenting	Family members help the children learn to be independent	Very dissatisfied	0 (0.0%)
		Dissatisfied	7 (6.9%)
		Satisfied nor dissatisfied	10 (9.8%)
		Satisfied	55 (53.9%)
		Very satisfied	30 (29.4%)
	Family members help the children with schoolwork and activities	Very dissatisfied	0 (0.0%)
		Dissatisfied	2 (2.0%)
		Satisfied nor dissatisfied	11 (10.8%)
		Satisfied	59 (57.8%)
		Very satisfied	30 (29.4%)
	Family members teach the children how to get along with others	Very dissatisfied	0 (0.0%)
		Dissatisfied	3 (2.9%)
		Satisfied nor dissatisfied	10 (9.8%)
		Satisfied	54 (52.9%)
		Very satisfied	35 (34.3%)
	Adults in my family teach the children to make good decisions	Very dissatisfied	2 (2.0%)
		Dissatisfied	6 (5.9%)
		Satisfied nor dissatisfied	16 (15.7%)
		Satisfied	53 (52.0%)
		Very satisfied	25 (24.5%)
	Adults in my family know other people in the children's lives (i.e. friends, teachers)	Very dissatisfied	1 (1.0%)
		Dissatisfied	10 (9.8%)
		Satisfied nor dissatisfied	16 (15.7%)
		Satisfied	50 (49.0%)
		Very satisfied	25 (24.5%)
	Adults in my family know how to spend time and care for each child's needs in the family	Very dissatisfied	4 (3.9%)
		Dissatisfied	12 (11.8%)
		Satisfied nor dissatisfied	13 (12.7%)
		Satisfied	52 (51.0%)
		Very satisfied	21 (20.6%)
Emotional Well-being	My family has the support we need to relieve stress	Very dissatisfied	12 (11.8%)
		Dissatisfied	9 (8.8%)
		Satisfied nor dissatisfied	15 (14.7%)
		Satisfied	49 (48.0%)
		Very satisfied	17 (16.7%)
	My family members have friends or others who provide support	Very dissatisfied	14 (13.7%)
		Dissatisfied	15 (14.7%)
		Satisfied nor dissatisfied	18 (17.6%)
		Satisfied	37 (36.3%)
		Very satisfied	18 (17.6%)
	My family members have some time to pursue their own interests	Very dissatisfied	9 (8.8%)
		Dissatisfied	13 (12.7%)
		Satisfied nor dissatisfied	26 (25.5%)
		Satisfied	39 (38.2%)
		Very satisfied	15 (14.7%)
	My family has outside help available to us to take care of the special needs of all	Very dissatisfied	17 (16.7%)
		Dissatisfied	22 (21.6%)
		Satisfied nor dissatisfied	27 (26.5%)
		Satisfied	25 (24.5%)
		Very satisfied	11 (10.8%)
Physical / Material Well-being	My family members have transportation to get to the places they need to be	Very dissatisfied	4 (3.9%)
		Dissatisfied	8 (7.8%)
		Satisfied nor dissatisfied	11 (10.8%)
		Satisfied	45 (44.1%)
		Very satisfied	34 (33.3%)
	My family gets dental care when needed	Very dissatisfied	15 (14.7%)
		Dissatisfied	18 (17.6%)
		Satisfied nor dissatisfied	15 (14.7%)
		Satisfied	33 (32.4%)
		Very satisfied	21 (20.6%)
	My family gets medical care when needed	Very dissatisfied	6 (5.9%)
		Dissatisfied	11 (10.8%)
		Satisfied nor dissatisfied	11 (10.8%)
		Satisfied	41 (40.2%)
		Very satisfied	33 (32.4%)
	My family has a way to take care of our expenses	Very dissatisfied	9 (8.8%)
		Dissatisfied	14 (13.7%)
		Satisfied nor dissatisfied	14 (13.7%)
		Satisfied	43 (42.2%)
		Very satisfied	22 (21.6%)
	My family feels safe at home, work, school, and in our neighborhood	Very dissatisfied	4 (3.9%)
		Dissatisfied	3 (2.9%)
		Satisfied nor dissatisfied	9 (8.8%)
		Satisfied	49 (48.0%)
		Very satisfied	37 (36.3%)
Disability-Related Support	My family member with special needs has support to make progress at school or workplace	Very dissatisfied	11 (10.8%)
		Dissatisfied	18 (17.6%)
		Satisfied nor dissatisfied	17 (16.7%)
		Satisfied	28 (27.5%)
		Very satisfied	28 (27.5%)
	My family member with special needs has support to make progress at home	Very dissatisfied	5 (4.9%)
		Dissatisfied	8 (7.8%)
		Satisfied nor dissatisfied	14 (13.7%)
		Satisfied	47 (46.1%)
		Very satisfied	28 (27.5%)
	My family member with special needs has support to make friends	Very dissatisfied	10 (9.8%)
		Dissatisfied	19 (18.6%)
		Satisfied nor dissatisfied	24 (23.5%)
		Satisfied	34 (33.3%)
		Very satisfied	15 (14.7%)
	My family member has a good relationship with the service providers who work with our family member with a disability	Very dissatisfied	7 (6.9%)
		Dissatisfied	13 (12.7%)
		Satisfied nor dissatisfied	14 (13.7%)
		Satisfied	42 (41.2%)
		Very satisfied	26 (25.5%)

Factors associated with satisfaction

The survey compared various parameters concerning satisfaction levels. For centers, the "online" mode showed the highest satisfaction at 49.4%, whereas "KAMC Riyadh" followed closely with 28.7%. When broken down by age groups, there was not a significant disparity, as the 8-10 and 11-13 age groups both stood at around 26.4%. Males dominated the satisfaction levels at 73.6%, in contrast to females at 26.4%; however, there was no statistically significant difference. In terms of severity scores, Level 2 and Level 1 were comparable, with 48.3% and 41.4%, respectively. Among ASD children's programs, satisfaction was fairly distributed, though speech and language therapy had a slight edge at 69%. A substantial portion of relatives, specifically in the 30-49 age group, reflected satisfaction, and those with a college degree represented a major portion at 59.8%. Regarding occupation, while the government sector had a higher satisfaction rate of 43.7%, the unemployed segment cannot be overlooked at 27.6%. Nationality-wise, Saudis were more represented with 85.1% satisfaction. The majority of respondents fell under the monthly income group of less than 10,000 SAR, capturing 57.5%. Lastly, mothers took the lead in the relationship category with 57.5%. Importantly, all parameters had p-values above 0.05, highlighting that the differences in satisfaction across these categories were not statistically significant, as shown in Table 3.

**Table 3:** Factors associated with satisfaction among patients' families SAR: Saudi Arabia Riyal; TEACCH: Treatment and Education of Autistic and Related Communication Handicapped Children; ASD: autism spectrum disorder; KAMC: King Abdulaziz Medical City; ABA: applied behavior analysis

Parameters		Poor satisfaction	Satisfied	p-value
Center	KAMC Riyadh	4 (26.7%)	25 (28.7%)	0.497
	KAMC Jeddah	4 (26.7%)	10 (11.5%)	
	Online	6 (40.0%)	43 (49.4%)	
	Others	1 (6.7%)	9 (10.3%)	
Age group	1-4 years	2 (13.3%)	10 (11.5%)	0.830
	5-7 years	2 (13.3%)	18 (20.7%)	
	8-10 years	3 (20.0%)	23 (26.4%)	
	11-13 years	5 (33.3%)	18 (20.7%)	
	14-16 years	3 (20.0%)	18 (20.7%)	
Gender	Male	14 (93.3%)	64 (73.6%)	0.183
	Female	1 (6.7%)	23 (26.4%)	
Severity score	Level 1 (mild/requiring support)	4 (26.7%)	36 (41.4%)	0.192
	Level 2 (moderate/requiring substantial support)	7 (46.7%)	42 (48.3%)	
	Level 3 (severe/requiring very substantial support)	4 (26.7%)	9 (10.3%)	
ASD children program	ABA therapy	3 (20.0%)	23 (26.4%)	0.775
	Behavioral therapy	8 (53.3%)	52 (59.8%)	0.778
	Speech and language therapy	11 (73.3%)	60 (69.0%)	1.00
	Occupational therapy	8 (53.3%)	36 (41.4%)	0.411
	Sensory integration therapy	4 (26.7%)	16 (18.4%)	0.486
	Special education programs	5 (33.3%)	34 (39.1%)	0.779
	TEACCH	4 (26.7%)	14 (16.1%)	0.297
Parents age	<30	1 (6.7%)	10 (11.5%)	0.903
	30-39	6 (40.0%)	32 (36.8%)	
	40-49	5 (33.3%)	32 (36.8%)	
	50-59	3 (20.0%)	11 (12.6%)	
	≥60	0 (0.0%)	2 (2.3%)	
Educational level	High school	3 (20.0%)	26 (29.9%)	0.704
	Higher education	2 (13.3%)	9 (10.3%)	
	College	10 (66.7%)	52 (59.8%)	
Occupation	Unemployed	8 (53.3%)	24 (27.6%)	0.121
	Government sector	3 (20.0%)	38 (43.7%)	
	Private sector	2 (13.3%)	16 (18.4%)	
	Student	1 (6.7%)	1 (1.1%)	
	Retired	1 (6.7%)	8 (9.2%)	
Nationality	Saudi	11 (73.3%)	74 (85.1%)	
	Non-Saudi	4 (26.7%)	13 (14.9%)	0.270
Monthly income	<10,000 SAR	9 (60%)	50 (57.5%)	0.231
	10,000-20,000 SAR	3 (20.0%)	30 (34.5%)	
	>20,000 SAR	3 (20.0%)	7 (8.0%)	
Relation	Mother	11 (73.3%)	50 (57.5%)	0.675
	Father	4 (26.7%)	30 (34.5%)	
	Siblings	0 (0.0%)	2 (2.3%)	
	Other	0 (0.0%)	5 (5.7%)	

## Discussion

The current cross-sectional study evaluated the social context of autistic children in Saudi Arabia and the QOL for their families using the BCFQOL. The results offer a comprehensive perspective on how autism affects not just the diagnosed children but also their families. Notably, the majority of autistic children were male, aligning with global trends, which have consistently reported a higher prevalence of ASD in males than females [23,24]. The severity of autism, as observed in our study, reflects the distribution noted in other studies in the Middle East [25] and internationally [26].

Our findings indicated a comprehensive spectrum of therapeutic interventions employed for children with ASD in the surveyed population. Speech and language therapy emerged as the most prevalent intervention, utilized by nearly 70% of participants, underscoring the critical role it plays in addressing the communication challenges inherent in many autism diagnoses [27]. The second most adopted approach was behavioral therapy (58.8%), emphasizing the importance of behavioral interventions in managing and improving maladaptive behaviors and skill deficits. Occupational therapy, availed of by 43.1% of the respondents, also holds significance as it aids children in refining their daily living skills and sensory processing abilities. Additionally, while ABA therapy was accessed by a quarter of the participants, its evidence-based nature in effectively enhancing positive behaviors and decreasing negative ones in individuals with ASD can't be overlooked. Sensory integration therapy, special education programs, and TEACCH each cater to the specific needs of ASD individuals, be they sensory challenges, educational adaptations, or structured teaching. The majority of the study population receiving multiple therapeutic interventions suggests the complex and multifaceted nature of ASD, where a single mode of therapy often does not address all presenting challenges. Additionally, this trend highlights the recognition among caregivers and professionals of the need for a holistic and integrated approach to treatment, combining different therapies to ensure comprehensive care and optimize outcomes for children with ASD [28].

In terms of satisfaction scores based on BCFQOL, the family interaction and parenting domains showed higher satisfaction compared to other domains. These findings are quite different from those of Schlebusch et al., who investigated the QOL of families of South African ASD patients [29]. They showed that families were the most satisfied with the disability-related support that they were receiving. Other studies showed that families were most satisfied with their physical/material well-being [30-32]. On the other hand, some studies reported that families were the least satisfied with their emotional well-being [31,33,34]. The emotional well-being of families is a pivotal area that demands attention. As Meral et al. emphasized, emotional support plays a vital role in bolstering psychosocial health, mitigating stress, and advancing a positive life outlook [35]. The need to either enhance existing services or create new ones that specifically address the emotional needs of families is evident. While many families usually find support through informal channels like friends and relatives, there are instances where these informal networks may not be sufficient, necessitating professional intervention.

When compared against global data, the satisfaction scores derived from BCFQOL in this study were commendably high. The support frameworks, both globally and regionally, vary considerably, yet families in Saudi Arabia appear to have a relatively positive experience. In a study by Davis and Carter, family QOL was reported to be significantly lower among families with autistic children when compared to those with typically developing children [36]. However, our results indicated that a significant majority of families felt satisfied with family interactions and the support they received. This resonates with another study conducted in neighboring Jordan, which highlighted family cohesiveness and strong cultural support systems as paramount in enhancing QOL [37].

Clinical implications

The findings have several clinical implications. First, given the high satisfaction scores associated with speech and language therapy, healthcare providers should consider prioritizing it in the early intervention stages for autistic children in the region. Second, while emotional well-being and disability-related support had satisfactory scores, there remains an evident gap, suggesting the need for strengthened psychological and specialized support.

Future directions

Future research should delve deeper into specific factors contributing to the high satisfaction scores in the Saudi context. Understanding the unique cultural, social, and healthcare dynamics in the Saudi context is crucial, as these factors may significantly influence satisfaction scores regarding autism care. For example, when satisfaction questionnaires are translated, certain words might carry deeper religious connotations, potentially leading respondents to express satisfaction as a religiously appropriate response under various circumstances. Additionally, responses may be influenced by a lack of awareness about what optimal services entail. Therefore, investigating these cultural and social factors is essential to fully comprehend how they might affect the interpretation of results in autism care frameworks. Additionally, longitudinal studies can be valuable in tracking the QOL as autistic children transition to adulthood and the evolving challenges families might face.

Limitations

While our study offers significant insights, it is not devoid of limitations. The sample size is relatively small, which may hinder the generalizability of our findings. The reliance on self-reported questionnaires can introduce biases, and the cross-sectional nature of the study means causal relationships cannot be established. Additionally, the study predominantly reflects urban centers and online respondents, potentially not capturing the full spectrum of experiences across the country.

## Conclusions

This study offers a comprehensive understanding of the social context of autistic children in Saudi Arabia and the associated QOL for their families. The data indicates high satisfaction scores among Saudi families with autistic members, suggesting a perception of a supportive framework. However, this should not be conflated with the actual adequacy of services, and specific areas have been identified that require further enhancement. Emulating the positive aspects and addressing the gaps can significantly improve the global care landscape for autism.
